# ER stress-induced YAP upregulation leads to chondrocyte phenotype loss in age-related osteoarthritis

**DOI:** 10.3389/fphar.2024.1476255

**Published:** 2024-11-12

**Authors:** Yanchun Gao, Haifeng Wei, Xiaoyuan Peng, Chenchen Wang, Hongyi Zhu, Junhui Yin

**Affiliations:** ^1^ Department of Orthopaedic Surgery, Shanghai Sixth People’s Hospital Affiliated to Shanghai Jiao Tong University School of Medicine, Shanghai, China; ^2^ Department of Orthopedics Surgery, Shanghai Sixth People's Hospital Affiliated to Shanghai Jiao Tong University School of Medicine, Shanghai, China

**Keywords:** er stress, YAP, ctgf, pamrevlumab, degeneration, chondrocyte

## Abstract

**Background:**

Osteoarthritis (OA) is a common degenerative joint disease, leading to pain and restricted mobility. Age-related endoplasmic reticulum (ER) stress has been implicated in the pathogenesis of OA, but the underlying mechanisms remain unclear. This study aims to explore the relationship between age-related ER stress, YAP overexpression, and chondrocyte phenotype loss in the development of OA.

**Methods:**

Cartilage samples were collected from patients undergoing amputation, and age-related ER stress markers and YAP expression were assessed using immunohistochemical staining and qPCR. Transgenic mice with cartilage-specific YAP overexpression (YAP^OE^) were created, and Pamrevlumab was administered to evaluate its therapeutic effects.

**Results:**

Higher expression of ER stress markers and YAP were showed in aged tissues compared to younger tissues. YAP overexpression led to decreased levels of cartilage phenotype markers and increased osteogenesis-related proteins. *In vivo*, YAP^OE^ mice exhibited OA-like cartilage degeneration, which was mitigated by Pamrevlumab treatment.

**Conclusion:**

Age-related ER stress induces YAP overexpression, contributing to OA pathogenesis. Pamrevlumab effectively prevents this phenotype loss in YAP^OE^ mice, suggesting its potential as a therapeutic agent for OA. These findings provide new insights into the molecular mechanisms of OA and highlight the importance of targeting the ER stress-YAP-CTGF signaling pathway in OA treatment and prevention.

## 1 Introduction

Osteoarthritis (OA) is the most common joint disease in adults worldwide, characterized by the degenerative destruction of articular cartilage. The pathological changes in OA-affected joints manifest as pain and restricted mobility, leading to a significant decline in the quality of life for those affected (Litwic et al., 2013; Martel-Pelletier et al., 2016). The financial and social burdens induced by OA are increasingly severe, particularly in the older population (Adebayo et al., 2017). Despite treatments such as joint replacement and specific medications, the pathogenesis of OA remains unclear (Baldini et al., 2015; Marshall et al., 2014; McAlindon et al., 2014).

Age is a significant factor influencing the prevalence of knee and hip osteoarthritis in the elderly (Prieto-Alhambra et al., 2014; Long et al., 2022; GBD, 2021 Other Musculoskeletal Disorders Collaborators, 2023). Data from the Johnson County Osteoarthritis Project showed that the prevalence of radiographic knee osteoarthritis increased from 26.2% in the 55–64 age group to nearly 50% in the 75+ age group. Similarly, the prevalence of symptomatic hip osteoarthritis rose from 5.9% in the 45–54 age group to 17% in the 75+ age group (Ambrose et al., 2024). While many studies have focused on clinical evidence of age as a factor in OA prevalence, further research is needed to elucidate the pathogenesis of age-induced osteoarthritis (Hou et al., 2018; Liu et al., 2023). Age-related activation of endoplasmic reticulum (ER) stress is strongly associated with the development of several age-related diseases (Liu et al., 2018; Taylor, 2016; Pinky et al., 2023; Garcia et al., 2023).

Age-related activation of endoplasmic reticulum (ER) stress is strongly associated with the development of several age-related diseases (Liu et al., 2018; Taylor, 2016; Pinky et al., 2023; Garcia et al., 2023). Numerous studies have described a link between ER stress and OA (Li et al., 2016; Takada et al., 2011; Rellmann et al., 2021). In chondrocytes from OA patients of different ages, there is evidence of age-dependent ER stress and the unfolded protein response (UPR). Aged articular cartilage chondrocytes exhibit decreased expression of calnexin and increased immunohistochemical staining for ER stress markers, suggesting that reduced expression of molecular chaperones during aging induces ER stress (Tan et al., 2020). Recent research has indicated that ER stress can contribute to various diseases through the activation of the YAP pathway (Liu et al., 2021; Panda et al., 2022; Anerillas et al., 2023; Wang et al., 2022).

The Hippo/YAP pathway is critically involved in cartilage development and plays a key role in cell fate and tissue regeneration (Yang et al., 2017; Hansen et al., 2015), and finally leads to Osteoarthritis (Sun et al., 2023). Increasing evidence has indicated that the Hippo/YAP pathway is also tightly involved in cartilage development (Karystinou et al., 2015), YAP was found as a negative regulator of chondrocyte differentiation as it promotes early chondrocyte proliferation via binding TEADs for direct upregulation of Sox6 but inhibits subsequent chondrocyte hypertrophy and maturation (Deng et al., 2016). Connective tissue growth factor (CTGF), a significant downstream protein of YAP, is abundantly expressed in chondrocytes of patients with severe osteoarthritis (Shome et al., 2020; Dudek et al., 2021; MacDonald et al., 2021; Tang et al., 2018). It is suggested that the activity of YAP-CTGF is tightly related to the OA progression (Zhang et al., 2020).

This study aims to investigate the relationship between ER stress and YAP signaling in the context of cartilage phenotype loss in OA. By providing an in-depth analysis of the pathogenesis of osteoarthritis, we aim to propose a new direction for future research based on our findings.

## 2 Results

### 2.1 Age-related ER stress in chondrocytes

**FIGURE 1:**
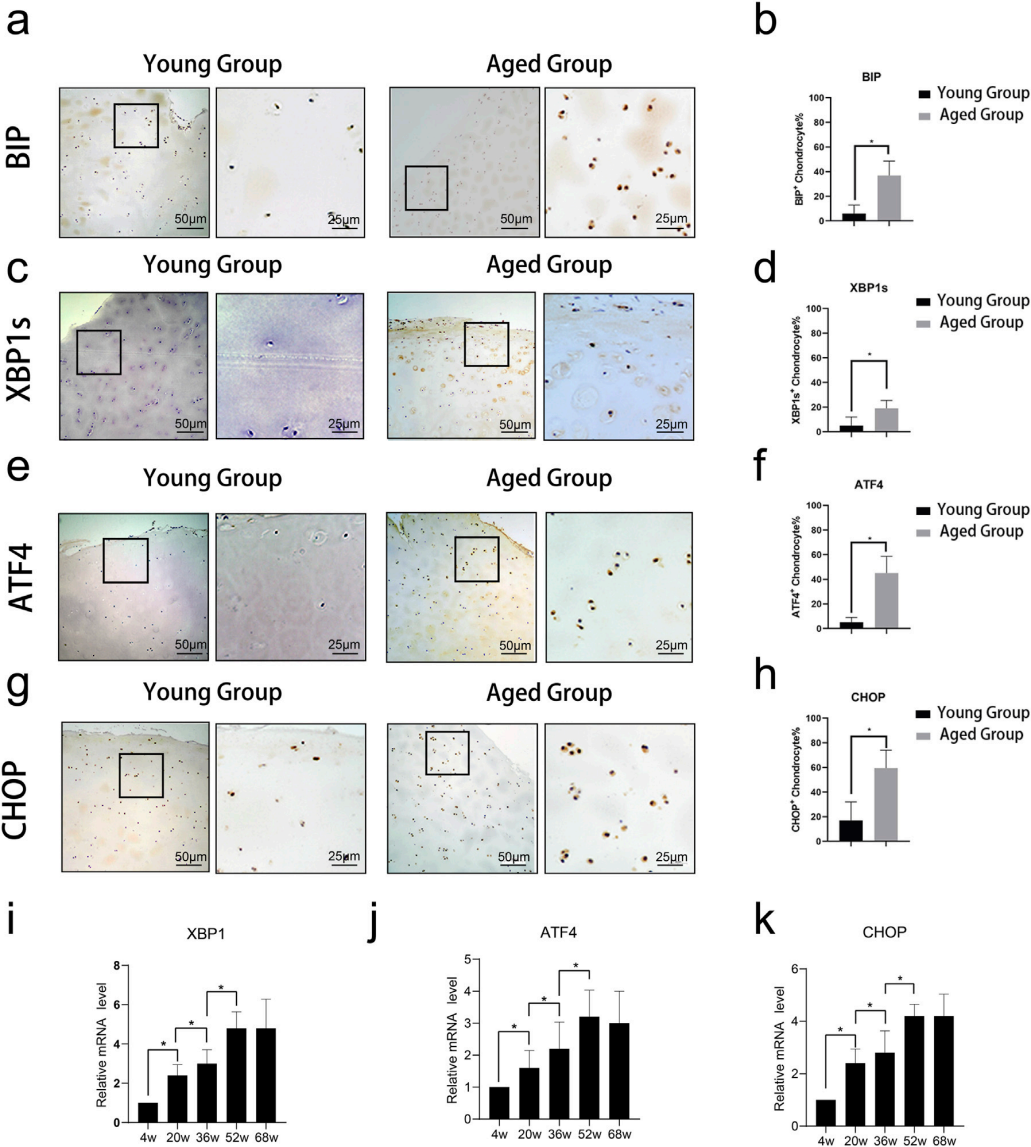
Expression of ER stress-related proteins in cartilage tissue across different age groups. Immunohistochemical staining of cartilage tissue revealed the expression of ER stress-related proteins BIP, XBP1s, ATF4, and CHOP. **(A–D)** Representative images of immunohistochemical staining for ER stress-related proteins BIP, XBP1s, ATF4, and CHOP in cartilage tissue from young (4w, 20w) and aged (52w, 68w) rats. Staining intensity and the number of positive cells were notably higher in the aged groups compared to the young groups, indicating increased expression of these proteins with age. **(E-H)** Quantitative analysis of the percentage of BIP, XBP1s, ATF4, and CHOP-positive chondrocytes in young and aged rats. Data shows significantly higher expression levels of all four proteins in the aged group (*p < 0.05). **(I-K)** Quantitative analysis of relative mRNA expression levels of XBP1s, ATF4, and CHOP in cartilage tissue from rats at 4, 20, 36, 52, and 68 weeks. The results demonstrate a gradual increase in mRNA expression with age, highlighting age-associated upregulation of ER stress-related gene expression. Statistical significance is denoted by *p < 0.05.

Immunohistochemical staining of cartilage tissue revealed the expression of ER stress-related proteins BIP, XBP1s, ATF4, and CHOP. Quantitative analysis of these results showed that ER stress-related proteins were highly expressed in the Aged Group (Figures 1A–H). Cartilage tissues were extracted from SD rats at different ages (4, 20, 36, 52, and 68 weeks) and subjected to qPCR. The results indicated that the mRNA expression levels of XBP1, ATF4, and CHOP increased with age (Figures 1I–K).

### 2.2 YAP expression and cartilage phenotype changes in aging

**FIGURE 2:**
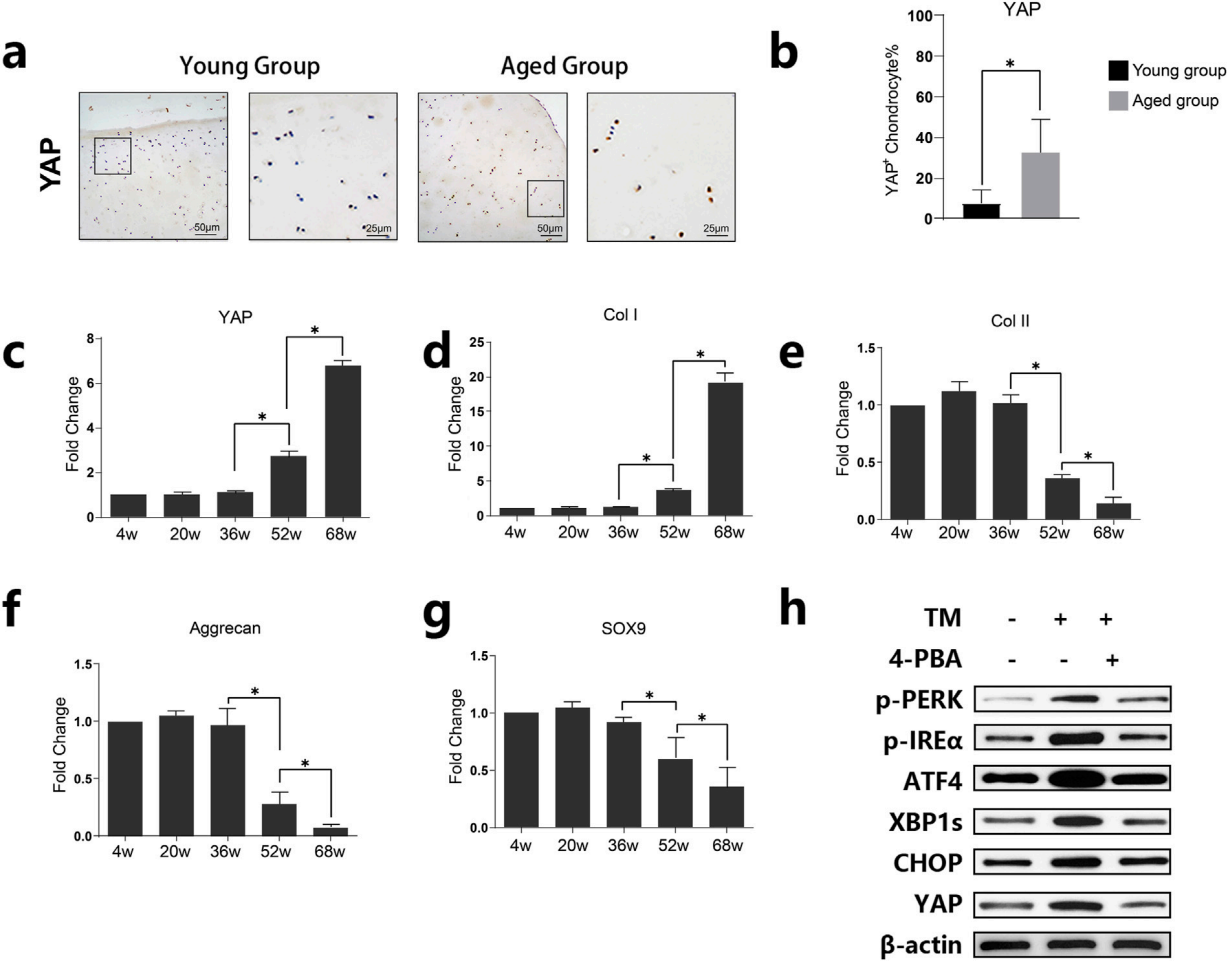
Expression of YAP and its association with age-related changes in cartilage tissue. **(A)** Representative images of immunohistochemical staining for YAP in cartilage tissues from young and aged groups. Increased staining in the aged group suggests higher YAP expression in older cartilage. **(B)** Quantitative analysis of YAP-positive chondrocytes in young and aged groups. Data are presented as mean ± SD, showing significantly elevated levels of YAP in the aged group (*p < 0.05). **(C–G)** Quantitative analysis of relative mRNA levels of YAP, Col I, Col II, Aggrecan, and SOX9 across different age groups (4w, 20w, 36w, 52w, 68w). The results show age-dependent changes, with YAP and Col I expression increasing, while Aggrecan and SOX9 decrease with age, indicating cartilage degeneration with age **(H)** Western blot analysis showing the expression levels of YAP, p-PERK, p-IREα, ATF4, XBP1s, and CHOP in the presence or absence of tunicamycin (TM) and 4-phenylbutyric acid (4-PBA). The results demonstrate ER stress upregulated YAP whereas inhibition of ER stress reduced YAP expression.

Immunohistochemical staining showed higher YAP expression in the Aged Group (Figures 2A,B). qPCR analysis of cartilage from rats of different ages demonstrated that mRNA expression levels of YAP and Col I increased with age (Figures 2C,D), whereas the expression levels of Col II, Aggrecan, and SOX9 decreased, indicating a degenerative cartilage phenotype with age (Figures 2E–G). Human chondrocytes were treated with TM (an ER stress inducer) and 4-PBA (an ER stress inhibitor). As demonstrated by Western blot analysis TM induces endoplasmic reticulum (ER) stress, leading to increased phosphorylation of PERK and IRE, and elevated expression of ER stress-related proteins such as ATF4, XBP1s, and CHOP. In contrast, 4-PBA can mitigate these changes. Correspondingly, ER stress activation results in the upregulation of YAP, while inhibition of ER stress reduces YAP expression (Figure 2H).

### 2.3 Overexpression of YAP leads to cartilage phenotype loss and upregulation of osteogenesis-related proteins

**FIGURE 3:**
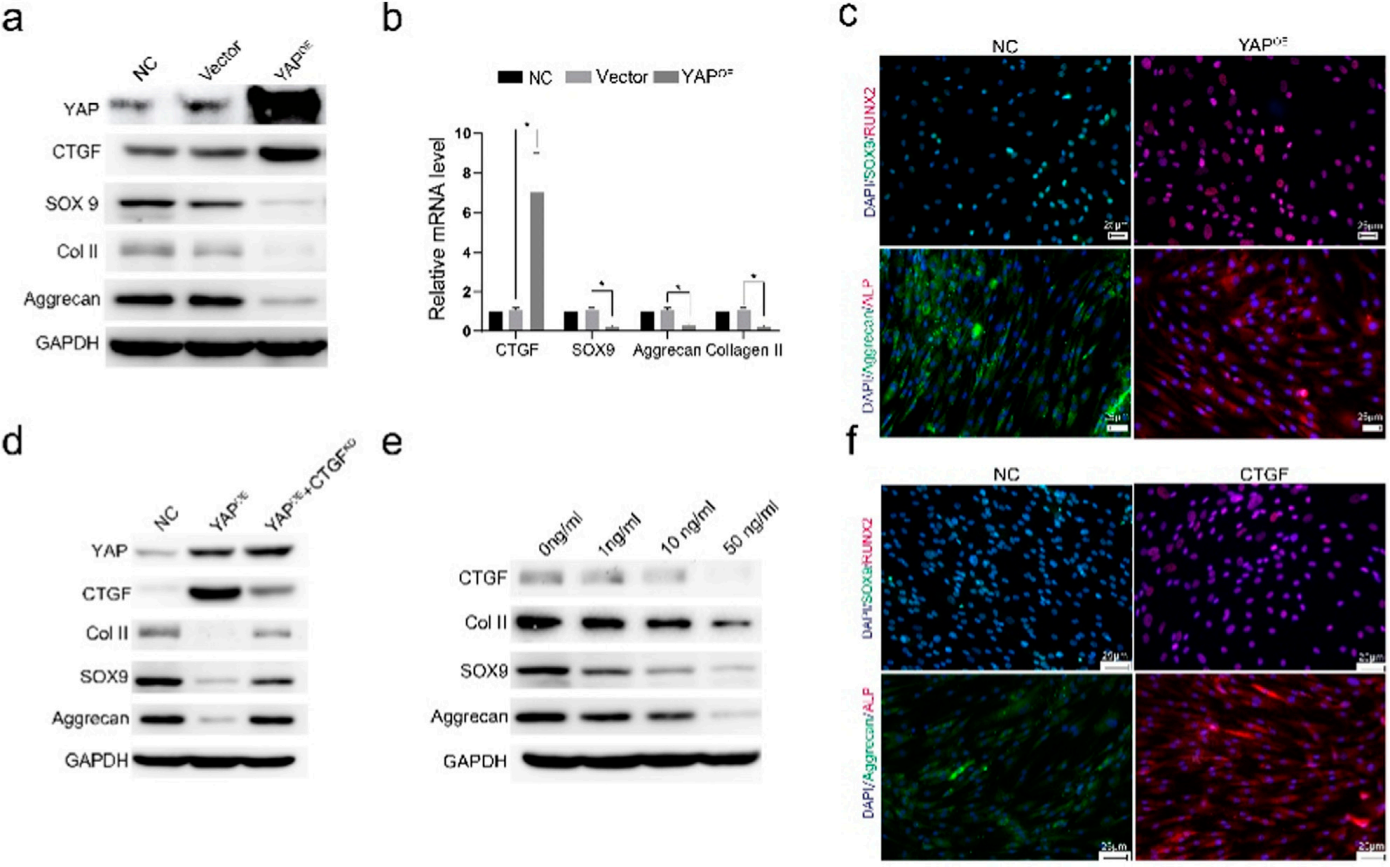
YAP overexpression leads to cartilage phenotype loss and upregulation of osteogenic-related proteins. **(A)** Western blot analysis showing the expression levels of YAP, CTGF, SOX9, COL II, and Aggrecan in cells with YAP overexpression. The results indicate that YAP overexpression promotes the upregulation of CTGF and downregulation of cartilage-specific proteins. **(B)** Quantitative analysis of mRNA expression levels of CTGF, SOX9, Aggrecan, and COL II under different treatment conditions. Data suggest a significant increase in CTGF and a reduction in cartilage markers with YAP overexpression (*p < 0.05). **(C)** Immunofluorescence staining for SOX9 and Aggrecan (cartilage markers), and ALP and RUNX2 (osteogenesis-related proteins) in control (NC) and YAP-overexpressing (YAP^OE^) groups. YAP overexpression decreases cartilage markers while increasing osteogenesis-related markers, indicating a shift toward an osteogenic phenotype. **(D)** Western blot analysis of COL II, SOX9, and Aggrecan in different groups, including NC, YAP^OE^), and YAP^OE^ with CTGF knockdown (YAP^OE^ + CTGF^KD^). The results show that CTGF knockdown partially rescues the cartilage phenotype loss induced by YAP overexpression. **(E)** Western blot analysis of COL II, SOX9, and Aggrecan treated with varying concentrations of CTGF (0, 1, 10, 50 ng/mL). Increasing CTGF concentrations leads to the reduction of cartilage markers, supporting its role in the loss of cartilage phenotype. **(F)** Immunofluorescence staining of SOX9/Aggrecan (cartilage markers) and ALP/RUNX2 (osteogenesis-related proteins) with or without CTGF stimulation. The presence of CTGF maintains cartilage markers and reduces the expression of osteogenesis-related markers, indicating its involvement in the cartilage-to-bone phenotype transition.

Overexpression of YAP in chondrocytes via lentivirus transfection resulted in increased expression of CTGF and decreased levels of SOX9, Col II, and Aggrecan, as shown by Western blot (Figure 3A). qPCR analysis showed similar trends (Figure 3B). Immunofluorescent staining indicated decreased SOX9 and Aggrecan and increased osteogenesis-related proteins ALP and RUNX2 in YAP-overexpressing chondrocytes (Figure 3C. These *in vitro* results suggest that increased YAP expression leads to the loss of cartilage phenotype and upregulation of osteogenesis-related proteins.

Western blot analysis showed that CTGF knockdown rescued the reduction of Col II, SOX9, and Aggrecan in YAP-overexpressing chondrocytes (Figure 3D). Treatment with varying concentrations of CTGF led to decreased expression of Col II, Aggrecan, and SOX9 (Figure 3E). Immunofluorescent staining confirmed the downregulation of SOX9 and Aggrecan and upregulation of osteogenesis-related proteins ALP and RUNX2 after CTGF stimulation (Figure 3F). Increased CTGF levels induced the loss of cartilage phenotypes and upregulation of osteogenic phenotypes.

### 2.4 YAP^OE^ induces osteoarthritis in a transgenic mouse model

**FIGURE 4:**
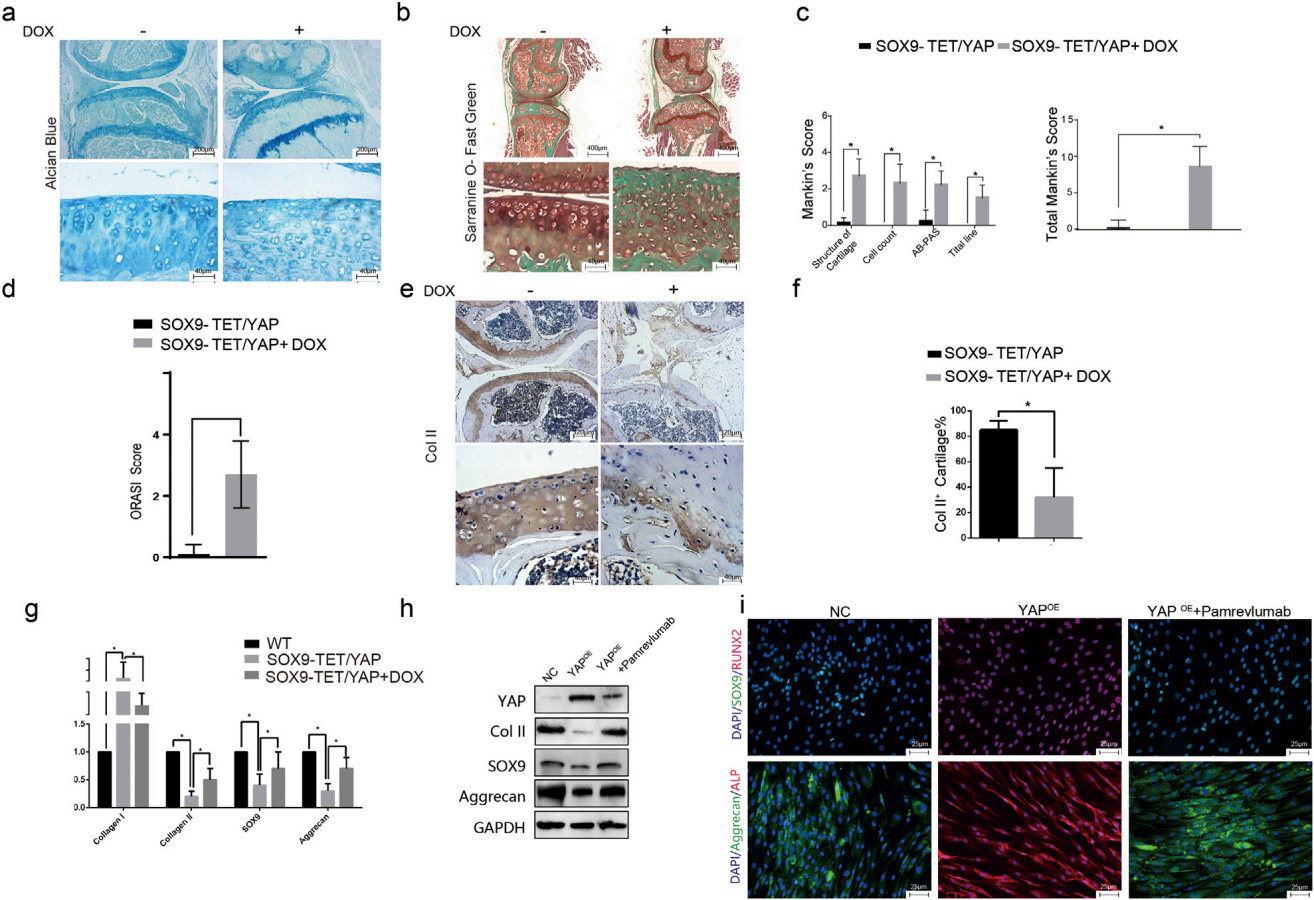
YAP overexpression induces osteoarthritis-like changes in a transgenic mouse model. **(A)** Representative images of Alcian blue staining of knee joints in transgenic mice with YAP^OE^. The staining indicates a reduction in cartilage matrix components in the YAP^OE^ mice, suggesting cartilage degradation. **(B)** Representative images of Safranin-O and Fast Green staining of knee joints in transgenic mice with YAP^OE^. The images show cartilage deterioration and decreased proteoglycan content in the presence of YAP overexpression. **(C–D)** Quantitative assessment using Mankin’s score and Osteoarthritis Research Society International (ORASI) score to evaluate cartilage damage in YAP^OE^ transgenic mice with or without DOX administration. YAP overexpression (induced by DOX) significantly increases cartilage degeneration scores (*p < 0.05). **(E–F)** Immunohistochemical staining and quantitative analysis of COL II in YAP^OE^ transgenic mice, showed decreased COL II expression in the YAP^OE^ group, indicating loss of cartilage integrity. **(G)** Relative mRNA expression levels of cartilage-associated genes (COL I, COL II, SOX9, and Aggrecan) across NC, YAP^OE^, and YAP^OE^ treated with Pamrevlumab groups. Pamrevlumab treatment partially restores cartilage gene expression, counteracting the effects of YAP overexpression. **(H)** Western blot analysis showing the protein levels of YAP, COL II, SOX9, and Aggrecan in control and YAP^OE^ mice, with and without Pamrevlumab treatment. The data indicate that Pamrevlumab reduces YAP-induced downregulation of cartilage markers. **(I)** Immunofluorescence staining for cartilage-specific proteins (SOX9, Aggrecan) and osteogenesis-related markers (ALP, RUNX2) in control, YAP^OE^, and YAP^OE^ + Pamrevlumab-treated groups. Pamrevlumab treatment appears to mitigate the YAP-induced increase in osteogenic markers, supporting its protective role against cartilage degradation.

Overexpression of YAP in cartilage-specific transgenic mice resulted in irregular cartilage morphology and decreased cartilage thickness, as shown by Alcian blue and Safranin-O and Fast green staining (Figures 4A,B). Mankin’s Score and ORASI Score were significantly higher in the YAP^OE^ group compared to the control, indicating a stronger tendency toward osteoarthritis (Figures 4C,D). Immunohistochemical staining showed lower Col II and higher CTGF expression, indicating cartilage phenotype loss following YAP activation (Figures 4E,F). A significant reduction in Col II + cells and an increase in CTGF + chondrocytes were observed in the YAP^OE^ group (Figure 4G). These results demonstrate that YAP overexpression induces osteoarthritis in the transgenic mice model.

### 2.5 Pamrevlumab rescued YAP overexpression-induced cartilage phenotype loss in vitro

Pamrevlumab treatment rescued the downregulation of Col II, SOX9, and Aggrecan caused by YAP overexpression, as shown by Western blot (Figure 4H). Immunofluorescent staining confirmed that Pamrevlumab rescued the reduction of SOX9 and Aggrecan, while osteogenesis-related proteins ALP and RUNX2 decreased (Figure 4I). Thus, Pamrevlumab can rescue YAP^OE^-induced chondrocyte phenotype loss.

### 2.6 Amrevlumab rescues YAP overexpression-induced osteoarthritis in vivo

**FIGURE 5:**
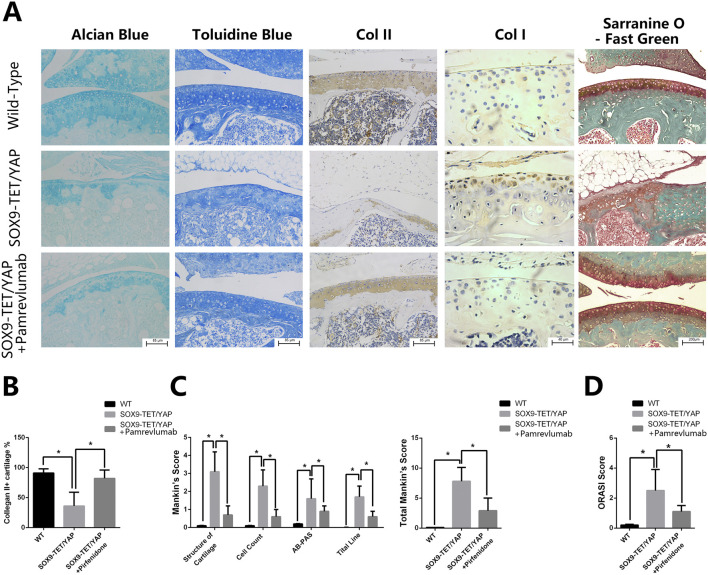
Pamrevlumab rescued YAP overexpression-induced osteoarthritis *in vivo*. **(A)** Representative images of chemical staining (Alcian Blue, Toluidine Blue, and Safranin O-Fast Green) from cartilage tissue of Wild-Type (WT), YAP^OE^, and YAP^OE^ + Pamrevlumab-treated mice. The YAP^OE^ group exhibits significant cartilage degradation, while Pamrevlumab treatment improves cartilage integrity, resembling the WT condition. **(B)** Immunohistochemical staining for COL II and COL I in cartilage tissues from WT, YAP^OE^, and YAP^OE^ + Pamrevlumab groups. The results show reduced COL II and increased COL I expression in the SOX9-TET/YAP group, indicative of cartilage loss and osteoarthritis, whereas Pamrevlumab treatment partially restores COL II levels. **(C)** Quantitative analysis of COL II-positive cartilage percentage across the three groups. Pamrevlumab significantly increases COL II presence compared to untreated SOX9-TET/YAP mice (*p < 0.05). **(D)** Mankin’s score analysis of cartilage structure, cell count, AB-PAS staining, and tidemark integrity. The YAP^OE^ group shows elevated scores (indicative of cartilage degradation), while Pamrevlumab reduces these scores, suggesting cartilage preservation. **(E)** Osteoarthritis Research Society International (ORASI) score comparing WT, YAP^OE^, and YAP^OE^ + Pamrevlumab mice. Pamrevlumab significantly lowers ORASI scores in YAP overexpression models, demonstrating its potential protective effect against YAP-induced osteoarthritis.

Intra-articular injection of Pamrevlumab into YAPOE-induced mice showed a protective effect, as demonstrated by chemical staining with Alcian blue, Toluidine blue, and Safranin-O and Fast green (Figure 5A). Pamrevlumab treatment rescued the low expression of Col II and high expression of Col I observed in YAPOE mice (Figure 5B). The percentage of Collagen II + cells increased in Pamrevlumab-treated transgenic mice (Figure 5C). Mankin’s score and ORASI Score were significantly lower in Pamrevlumab-treated YAP^OE^ mice (Figures 5D,E). These findings illustrate that YAP-CTGF signaling regulates osteoarthritis pathogenesis and that Pamrevlumab can mitigate YAP^OE^-induced osteoarthritis.

## 3 Discussion

In this study, using knee cartilage samples from rats and humans of different ages, we demonstrated that age-related ER stress-induced YAP overexpression leads to the loss of chondrocyte phenotypes. After confirming through *in vitro* experiments that YAP overexpression is a key factor in chondrocyte phenotype loss, we developed a transgenic mouse model with YAP overexpression specifically in the knee joint. This mouse model spontaneously exhibited signs of osteoarthritis without requiring osteoarthritis induction. Through both *in vivo* and *in vitro* experiments, Pamrevlumab was proven effective in preventing osteoarthritis. We propose a novel conclusion that aging-induced ER stress activation triggers YAP-CTGF signaling, causing chondrocyte phenotype loss and leading to osteoarthritis. This conclusion offers a new research direction in the pathogenesis of osteoarthritis for future studies.

ER stress is involved in the development of several aging-related diseases and is an important target for the study of degenerative diseases (Chen et al., 2023; Gao et al., 2018). Researchers have pointed out that age-related increases in ER stress are significant triggers for the development of osteoarthritis (Dreier et al., 2022). Several studies have suggested that activation of ER stress is involved in the pathophysiologic process of osteoarthritis by causing chondrocyte death (Li et al., 2016; Rellmann et al., 2021; Tan et al., 2020; Yang et al., 2020). Furthermore, as an essential physiological process regulating cellular function, ER stress is involved in regulating various cellular phenotypes (Raines et al., 2022; He et al., 2021; Aghadi et al., 2022; Chen and Cubillos-Ruiz, 2021; Gao et al., 2020). Dibyendu K et al. pointed out that PERK-mediated phosphorylation of eIF2α drives the selective translation of ATF4 and CHOP, enhancing YAP expression (Panda et al., 2022). Wu et al. (Wu et al., 2015) demonstrated in a study on liver cancer that the PERK kinase-eIF2α axis can activate Yap, while prolonged ER stress can create a negative feedback loop to inhibit Yap and promote apoptosis. Another study showed that in gastric cancer, YAP may regulate ER stress by activating the ERK signaling pathway (Liu et al., 2019). Additionally, as a key downstream protein of the IRE1a signaling axis, XBP1s is also considered to participate in the regulation of the YAP/TAZ signaling pathway (Fan et al., 2020). In our study, we have identified for the first time the interaction between YAP activation and ER stress in osteoarthritis, which is completely different from previous research. Based on existing studies (Takaguri et al., 2017; Yap et al., 2021; Yap et al., 2020), we speculate that signaling molecules such as PERK and IRE1 must play a crucial role. Moving forward, we will continue to investigate the effects of the three classical downstream pathways of ER stress on the YAP signaling axis to clarify the specific mechanisms involved in osteoarthritis. Our study suggests that ER stress regulates chondrocyte phenotypes through the activation of the YAP pathway. Research on downstream signaling pathways following ER stress in chondrocytes is still ongoing.

The YAP signaling pathway plays an essential role in cell aging (Sladitschek-Martens et al., 2022). It is regarded as an age-related protein, upregulated in various cells, and is associated with a wide variety of degenerative diseases, including cardiogenesis, skeletal muscle aging, vascular senescence, macular degeneration, and hair cell regeneration (Monroe et al., 2019; Setiawan et al., 2021; Pan et al., 2021; Rudolf et al., 2020). Maintaining YAP function can rejuvenate old cells, while YAP overexpression can lead to a series of aging symptoms (Zhang et al., 2018; Cha et al., 2022). These studies confirm the correlation between the YAP pathway and age-related diseases. In this study, we also found a close correlation between YAP and aging in young and old human samples, as well as in rats of different ages, which is consistent with most research findings. Additionally, YAP-related proteins and pathways have been shown to have significant associations with osteoarthritis (OA) in numerous previous studies (Zhang et al., 2011). Recent studies indicate its critical role in the pathogenesis of osteoarthritis. Gong et al. found an evident association between increased total expression of YAP and degenerative cartilage from OA patients (Gong et al., 2019). YAP was markedly upregulated both in mRNA and protein levels in OA mice (Zhang et al., 2019). The activation of YAP is thought to cause the phenotypic loss of chondrocytes, contributing to osteoarthritis development. Conditional knockout of YAP in mice preserves collagen II expression and protects cartilage from degeneration in osteoarthritis models (Zhang et al., 2020). As a degenerative disease, osteoarthritis is closely related to endochondral bone formation, cartilage degeneration, and structural disorganization of subchondral bone (Cox et al., 2013; Hosaka et al., 2013; Saito et al., 2010). In osteoarthritis, MSCs show disordered chondrogenic differentiation. Recent studies found that chondrocytes and osteoblasts are not independent lineages. In the pathological process of osteoarthritis, hypertrophic chondrocytes survive the cartilage-to-bone transition and become osteogenic cells, explaining both cartilage degeneration and endochondral bone formation (Yang et al., 2014; Zhou et al., 2014; Staines et al., 2013).

CTGF is identified as a direct YAP target gene important for cell growth (Fan et al., 2013; Chan et al., 2011; Zhao et al., 2008). The YAP-CTGF signaling axis plays an indispensable role in endochondral ossification in osteoarthritis (Delve et al., 2020). Although research on this signaling axis in the field of osteoarthritis is relatively limited, it has been well studied in the fields of fibrosis and cancer, and may be mediated by TEAD (Qing et al., 2022; Li et al., 2022; Gabdulkhakova et al., 2023). According to previous research, when the Hippo pathway is activated, MST1/2 activates LATS1/2. The LATS1/2 kinase phosphorylates YAP at the Ser127 site. Phosphorylated YAP binds to 14-3-3 proteins, leading to its retention in the cytoplasm and preventing it from functioning as a transcriptional co-activator. Additionally, phosphorylated YAP is more prone to ubiquitination and degradation, which further reduces the level of active YAP. Thus, YAP phosphorylation plays a role in the negative regulation of YAP (Mannaerts et al., 2015; Li et al., 2024). YAP chondrocyte nuclear translocation is regulated by differential phosphorylation in response to anabolic and catabolic stimuli and contribute to reduced anabolic activity and promotion of further cartilage loss (Cui et al., 2023). Some studies have manipulated the YAP signaling axis by inhibiting or increasing YAP phosphorylation (Zhang et al., 2024). In this study, we used Pamrevlumab to directly inhibit CTGF, and therefore did not address the impact of YAP phosphorylation on this pathway.

Previous studies show its contribution to joint homeostasis and osteoarthritis by controlling matrix sequestration. The deletion of CTGF increased the thickness of the articular cartilage and protected mice from osteoarthritis (Tang et al., 2018). Pamrevlumab, a humanized anti-CTGF antibody, has been tested in clinical trials for muscular dystrophy, pancreatic cancer, liver fibrosis, idiopathic pulmonary fibrosis, and type 1 and 2 diabetes (Ramazani et al., 2018). In this study, Pamrevlumab was effective in preventing cartilage phenotype loss and osteoarthritis-like lesions caused by YAP overexpression. Despite the complex mechanism of osteoarthritis, it remains a potential medication for treating and preventing osteoarthritis (Kaplon and Reichert, 2021; Xie et al., 2021).

This study still has some limitations. First, we proposed that aging establishes a connection with the YAP-CTGF signaling axis through endoplasmic reticulum stress. However, it is undeniable that there are likely many interactions involving other signaling pathways. YAP is not the only protein associated with the development of osteoarthritis, but we believe our research introduces a new direction, providing a foundation for future basic research and targeted therapeutic drug development. Second, due to the limitations of experimental conditions, the sample size in the study is relatively small. A smaller sample size and manual analysis of images may lead to bias in the experimental results. However, we believe that these limited samples can still yield valid experimental results. In this study, we did not perform large-scale RNA sequencing analysis, But the results from existing literature related to RNA sequencing data support our view (Ji et al., 2019; Pemmari et al., 2020; Soul et al., 2018). Third, although the research on this drug shows promising prospects, there is still a long way to go before it can be applied clinically. Further exploration is needed regarding its long-term therapeutic effects, potential side effects, and optimal dosage.

## 4 Materials and methods

### 4.1 Patients and specimens

Human cartilage samples were obtained from 20 patients following amputation, as described by Battistelli (Battistelli et al., 2005). Patients were divided into two age groups: <50 years (young group) and >50 years (aged group) (Copp et al., 2023; Kim et al., 2024). This study was approved by the Ethics Committee of Shanghai Sixth People’s Hospital and complies with the Declaration of Helsinki.

### 4.2 Rats at different ages

All animal experiments were approved by the Animal Care and Use Committee of Shanghai Sixth People’s Hospital. Female SD rats of different ages (2 weeks, 18 weeks, 34 weeks, 50 weeks, 66 weeks) were obtained, with each age group containing five rats. They were maintained for 2 weeks, then euthanized, and cartilage tissues from their limbs were collected for subsequent PCR quantification experiments.

### 4.3 Establishment of the YAP^OE^ transgenic mice models and treatment

Ten mice were used: five wild-type and five YAP^OE^ transgenic mice. Wild-type C57BL/6 mice served as the control group. The gain-of-function transgenic Tet-On-YAP1 mice line was created by microinjection of C57BL/6 fertilized eggs with the pRP (Exp)-TRE3G > YAP1 mutation > IRES/EGFP plasmid. Transgene PCR primers were: Transgene PCR primer F: CGG​GGC​TAA​AGT​GCA​TCT​CG. Transgene PCR primer R: CCA​GGC​CAC​ATA​TGA​TTA​GTT​CCA​GG. The sequence of SOX9rtTA was obtained from the pRP (Exp)-sox9Promoter > Tet3G/IRES/tTS plasmid. Transgene PCR primer F: GGA​AGC​TGC​CCG​ACT​CCT​TCT​T. Transgene PCR primer R: CCT​GCC​ATG​TTG​TTG​TCT​GAT​CGA​TG. SOX9rtTA was crossed with Tet-On-YAP1 mice. To induce YAP expression in chondrocytes, eight-week-old SOX9rtTA/Tet-On-YAP1 mice were treated with 2 mg/mL doxycycline in drinking water for 8 weeks. This established a transgenic mice model overexpressing YAP in chondrocytes.

To verify the protective effect of Pamrevlumab in YAP^OE^ mice, 60 mice were included: 20 wild-type and 40 YAP^OE^ transgenic mice. 20 of the transgenic mice served as positive controls and 20 were treated with Pamrevlumab. In the negative and positive control groups, saline was injected intra-articularly and periarticularly twice a week from 2 weeks before to the end of doxycycline administration. The treatment group received Pamrevlumab (40 mg/kg) injections twice a week over the same period. Randomization was not used to allocate experimental units. No unscheduled animal deaths occurred before completing the experiments. Randomization was not used to allocate experimental units. No unscheduled animal deaths occurred before completing the experiments.

### 4.4 Cell culture, treatment

Primary chondrocytes were isolated from cartilage fragments dissected from the femoral heads, femoral condyles, and tibial plateaus of C57BL/6 mice or humans. Articular cartilage was cut into small pieces and digested with 0.25% trypsin at 37°C for 30 min. After washing three times with PBS, the pieces were digested with 0.25% collagenase II at 37°C for 8 h. The cell suspension was filtered using a 70 μm cell strainer and centrifuged at 1,000 rpm for 5 min to collect primary chondrocytes. The chondrocytes were plated at a density of 0.15 × 10^5 cells/mL in DMEM with penicillin (100 U/mL), streptomycin (100 μg/mL), and 10% fetal bovine serum (Gibco, Grand Island, NY, United States), and cultured at 37°C in a humidified 5% CO_2_ atmosphere. Pamrevlumab (Selleck, China) was administered at a concentration of 100 μM 2 days before lentiviral transfection.

### 4.5 Lentivirus transfection

For protein overexpression and knockdown, targeted sequences were designed (Gene Pharma, Shanghai, China). Lentiviral vectors for YAP and CTGF were constructed by inserting the genes’ coding sequences (CDSs) into the pLenti vector. Virus packaging was conducted according to the VSVG-delta 8.9 system. Target sites in human genes included: shYAP^OE^: 5′-CTC​AGG​ATG​GAG​AAA​TTT​A-3′. shCTGF^KD^: 5′-GCT​GAC​CTG​GAA​GAG​AAC​ATT-3′. After 72 h of infection, the infection efficiency was assessed through Western blot analysis.

### 4.6 Western blot analysis

Cells were harvested and lysed with cell lysis buffer supplemented with protease and phosphatase inhibitors (Sigma-Aldrich, St. Louis, MO) on ice for 15 min. Protein concentrations were diluted 1:5 with protein loading buffer (Transgen Biotech, Beijing, China). A total of 30 µg of protein was subjected to SDS-PAGE after denaturation at 95°C for 5 min. Cell lysates were analyzed on a 10% Tris-HCl gel under reducing conditions. Proteins were transferred to 0.22-μm PVDF membranes (MERCK, Darmstadt, Germany) and blocked with 5% non-fat dry milk at 4°C overnight. Membranes were incubated for 2 h at 37°C with anti-SOX9 (Abcam, Cambridge, MA), anti-YAP, anti-aggrecan, anti-collagen II (Col II), and anti-GAPDH (Cell Signaling Technology, Danvers, MA). Secondary antibodies (Cell Signaling Technology, Danvers, MA) were used for 1 h at 37°C. After washing with tris buffered saline with Tween, membranes were exposed to an ECL substrate in a darkroom and analyzed with the FluorChem M system (ProteinSimple, San Jose, CA, United States). Results were quantified using Quantity One software (Bio-Rad Laboratories, Hercules, CA, United States).

### 4.7 Quantitative real-time polymerase chain reaction (qRT-PCR)

Full-thickness cartilage was collected from the knees of various aged SD rats. After excising the subchondral bone, the cartilage was chopped and homogenized with Trizol (Transgen Biotech, Beijing, China). cDNA synthesis was performed using TransScript^®^ All-in-One First-Strand cDNA Synthesis SuperMix for qPCR (Transgen Biotech). qPCR was performed with TransStart^®^ Top Green qPCR SuperMix (Transgen Biotech), using β-actin for normalization.

### 4.8 Immunohistochemistry and immunohistochemistry staining

Knees and articular cartilage were harvested and fixed in 4% formaldehyde for 48 h. Femoral condyles were decalcified in EDTA for 28 days, then dehydrated in graded ethanol, and made transparent with xylene. Specimens were embedded in paraffin and sectioned into 5 μm slices. Toluidine blue staining evaluated cartilage degeneration and calcium deposition (Shepard and Mitchell, 1976; Sridharan and Shankar, 2012). Safranin-O and fast green staining assessed matrix proteoglycan and joint morphology. Osteoarthritis progression was evaluated using the modified Mankin’s score and OARSI system (Chen et al., 2023; Zhang et al., 2020). Immunohistochemistry was performed to evaluate ER stress and chondrocyte phenotype, using primary antibodies for type II collagen, type I collagen, BIP, XBP1s, ATF4, CHOP, YAP, and CTGF (all from Abcam, 1:100).

### 4.9 Statistical methods

One-way analysis of variance (ANOVA) was used to compare the means of multiple groups. Fisher’s exact test compared disease incidence between groups. Independent-sample t-tests compared means between two groups. Statistical analysis was conducted using SPSS 20.0 (IBM Corp, Armonk, NY, United States). P values <0.05 were considered statistically significant.

## 5 Conclusion

This study found that the YAP-CTGF axis, activated by age-related ER stress, causes the loss of the chondrocyte phenotype. We established a transgenic mice model with YAP overexpressed in cartilage and observed osteoarthritis-like pathological changes in the knee tissue of the mice. Pamrevlumab inhibited this loss of chondrocyte phenotype due to YAP overexpression and prevented osteoarthritis development *in vivo*. This study suggests an important role for ER stress-induced YAP upregulation in degenerative knee diseases and provides new possibilities for the pharmacological prevention of osteoarthritis.

## Data Availability

The original contributions presented in the study are included in the article/Supplementary Material, further inquiries can be directed to the corresponding authors.
